# Safety Evaluation of Curcumol by a Repeated Dose 28-Day Oral Exposure Toxicity Study in Rats

**DOI:** 10.3390/toxics11020114

**Published:** 2023-01-24

**Authors:** Zhaoxu Yang, Sheng Wang, Yawen Hong, Renhua Gai, Wenxiang Hong, Bingbing Tang, Chunqin Lin, Xiaomeng Wang, Qiaojing Wang, Chao Chen, Jiajia Wang, Qinjie Weng

**Affiliations:** 1 Center for Drug Safety Evaluation and Research, College of Pharmaceutical Sciences, Zhejiang University, Hangzhou 310058, China; 2 Innovation Institute for Artificial Intelligence in Medicine, College of Pharmaceutical Sciences, Zhejiang University, Hangzhou 310058, China

**Keywords:** curcumol, 28-day exposure, oral toxicity study, SD rats, formulation, histopathology

## Abstract

Curcumol, a natural product isolated from the traditional Chinese medicine *Rhizoma curcumae*, possesses various potential therapeutic values in many diseases. However, evidence of its toxicological profile is currently lacking. In this study, a repeated toxicity study of curcumol was conducted for the first time. SD rats were exposed to doses of 250, 500, 1000 mg/kg in a selected dose formulation for 28 days through oral administration. The potential toxic effects of curcumol on the blood system were observed and further validated in vivo and in vitro. Moreover, other hematology and biochemistry parameters as well as the weight of organs were altered, but no related histopathological signs were observed, indicating these changes were not regarded as toxicologically relevant. Our current findings provide a complete understanding of the safety profile of curcumol, which may contribute to its further study of investigational new drug application.

## 1. Introduction

In recent years, extracts and active components of traditional medicine have become the focus of the pharmaceutical and food industry due to their extensive pharmacological activities and application value. *Rhizoma curcumae*, a traditional Chinese medicine, has been well-known for its use in alleviating pain, removing blood stasis, and anticoagulation over thousands of years [1]. The essential oil extract of *Rhizoma curcumae* has shown good bioactivities in anti-bacterial and anti-inflammatory properties, and has already been approved as a therapeutic remedy for disorders by the National Medical Products Administration (NMPA) of China [2]. The essential oil of *Rhizoma curcumae* is a mixture with around 20 typical constituents including curcumol, β-elemene, curdione, isocurcumenol, and other Sesquiterpenes [3]. Due to the complex composition of the essential oil, the existing extraction process and quality control standards are still far away to meet the safety and efficacy criteria of clinical drugs [4].

Curcumol, a type of sesquiterpenoid isolated from *Rhizoma curcumae*, was a major active ingredient in its essential oil extracts and included in the quality control standards of essential oil extracts stipulated in the Chinese Pharmacopoeia. It is reported that curcumol shows great potential in anti-tumor, anti-liver fibrosis, anti-inflammatory, and anti-viral activities [1,5,6,7,8,9,10], attracting much attention on its pharmacological properties. Moreover, compared to the essential oil of *Rhizoma curcumae*, the quality of curcumol can be better controlled, indicating that curcumol is a promising drug candidate for clinical use. However, information on curcumol for the Investigational New Drug (IND) application for clinical investigations, such as manufacturing information and animal toxicology studies, is currently lacking. Currently, several studies have been conducted about the safety evaluation of different extracts of *Curcuma*, and curcumol is included in these extracts [11,12]. However, the other components in the extracts may interfere with evaluation of the in vivo toxicity for individual chemicals [13]. Thus, it is of great significance to study the toxicological profile of curcumol in vivo for a better understanding of its safety profile in further drug discovery.

In this study, we explored the toxicity potential of curcumol for the first time and conducted a repeated toxicity assay of curcumol in Sprague–Dawley (SD) rats exposed to different doses of curcumol for 28 days through oral administration. The toxicity effects of curcumol were evaluated by the clinical symptoms, body weight (b.w.) changes, food consumption, biochemical and hematological parameters, necropsy, organ weight ratios, and histopathology of various tissues. The results from the complete assessment of this repeated toxicity study would allow us to provide valuable information to establish a safe dose of curcumol for further drug discovery.

## 2. Materials and Methods 

### 2.1. Test Article and Chemicals

Curcumol (95% purity) was obtained from the Dilger Medical Co., Ltd. (Nanjing, China). Dose formulations were prepared with cosolvents. All dosing solutions are prepared daily before administration. Cosolvents, such as olive oil, 1,2-propylene glycol, Ethanol and Kolliphor HS15, were purchased from Sigma-Aldrich Corp. (St. Louis, MO, USA). 

### 2.2. Experimental Animals

Male and female SD rats (six weeks) were purchased from Zhejiang Vital River Laboratory Animal Technologies Co., Ltd. (Zhejiang, China). The animals were acclimatized in a laboratory environment for seven days before the experiment and all the experimental animals were in good health and did not have pathogen infection. All animals were given free pellet food and water and put on a 12 h light/dark cycle. The room temperature and humidity of animal rooms were maintained at 22 ± 3 °C and 50 ± 10%, respectively. All animal use and studies were performed in compliance with all relevant ethical regulations and were approved by the Institutional Animal Care and Use Committee (IACUC) at Zhejiang University (IACUC-s22-019). 

### 2.3. Dose Formulation Analysis and Pharmacokinetic Assay

Four different dose formulations of curcumol were prepared according to previous studies and practices. Formulation 1, 50% aqueous olive oil [14]; formulation 2, 50% aqueous 1,2-propylene glycol [15]; formulation 3, deionized water with 10% ethanol and 10% Kolliphor HS15; formulation 4, deionized water with 10 % ethanol and 10% 1,2-propylene glycol.

Male SD rats were divided into four groups randomly and four different formulations were administered orally (1000 mg/kg). Blood samples were collected from the tail vein at 0.25 h, 0.5 h, 1 h, 2 h, 4 h, 6 h, 8 h, 10 h, and 24 h after administration. The plasma was obtained from the centrifuged blood sample and stored at −80 °C for analysis. The following LC-MS/MS method was developed to determine curcumol in plasma by a Xevo TQ-S triple quadrupole MS/MS (Waters Corp., Milford, USA). The pharmacokinetics parameters were calculated with pharmacokinetic software DAS 3.0 with non-compartment analysis: T_max_, time to reach the maximum concentration; C_max_, the maximum concentration; AUC, area under the concentration–time curve; t_1/2_, the elimination half–life; CL, body clearance; Vz, apparent volume of distribution.

### 2.4. Repeated 28-Day Toxicity Study Design

Though no direct evidence of curcumol toxicity is available, the in vivo toxicity of *curcuma singularis rhizome* extract, including curcumol as one of its major components, has been assessed and 1000 mg/kg was selected as the highest dose in the subacute toxicity study [11]. According to the ICH guideline M3(R2), limit doses for sub-chronic toxicity studies of 1000 mg/kg/day for rodents and non-rodents are considered appropriate in most cases. Similar high-dose settings have also been reported in previous study [11]. Hence, we finally chose 1000 mg/kg as the high dose in the toxicity study. The low and medium dose were set at 250 and 500 mg/kg based on the recommendations of descending doses using a 2-fold interval factor in the guideline OECD 407 (2008), respectively. Furthermore, the low dose (250 mg/kg) was higher than the effective dose of curcumol (3~30 mg/kg i.g.) [16], which is sufficient to produce a therapeutic effect as well as pharmacokinetic exposure according to the Guideline on repeated dose toxicity - Revision 1 (EMA, 2010). Rats were weighed individually and randomly assigned to three dose groups and a control group (five rats/sex/group). Thus, experimental rats were administered with curcumol at the doses of 250, 500, and 1000 mg/kg b.w./day and control rats were treated with vehicle for continuous 28 days, and then rats were sacrificed for toxicity examination.

### 2.5. Clinical Observation, Body Weight and Food Consumption Recording

Each animal was observed before administration daily for clinical signs, including appearance, physical signs, skin, behavioral activities, glandular secretion, respiration, eyes, ears, nose, anus, fecal traits, limbs, and local reactions. The morbidity and mortality of each animal were recorded, including the time, extent, and duration of occurrence. The body weight of each animal was measured before the initiation and once a week after curcumol administration. The average body weight was calculated weekly for each group and each sex. The total food intake of each cage was recorded, and the average food consumption of each animal was calculated weekly.

### 2.6. Hematology and Biochemistry Analysis

Blood samples were extracted from the heart by an intracardiac injection under pentobarbital sodium anesthesia at day 29. Before blood samples collection, all animals were fasted overnight. Then hematology parameters were detected by an automated hematology analyzer Sysmex XT-2000i (Sysmex Corporation, Kobe, Japan): white blood cell count (WBC), red blood cell count (RBC), neutrophils (NEUT), lymphocytes (LYMPH), monocytes (MONO), eosinophils (EO), hemoglobin (HGB), hematocrit (HCT), mean corpuscular volume (MCV), mean corpuscular hemoglobin (MCH), mean corpuscular hemoglobin concentration (MCHC), blood platelet count (PLT). 

Biochemical parameters were measured on an automatic chemistry analyzer Cobas c311 (Roche Diagnostics, Basel, Switzerland): total protein (TP), albumin (ALB), alanine aminotransferase (ALT), aspartate aminotransferase (AST), total bilirubin (TB), alkaline phosphatase (ALP), blood urea nitrogen (BUN), glucose (GLU), triglyceride (TG), total cholesterol (TC), creatine kinase (CK), sodium (Na^+^), potassium (K^+^) and chloride (Cl^−^) ions. Hematological and biochemical parameters were included in the scope of detection according to the guidelines OECD 407 (2008).

### 2.7. Necropsy, Organ Weight and Histopathology

Before the sacrifice, animals were fasted overnight and then profoundly anesthetized with pentobarbital sodium. Animals were exsanguinated by intracardiac injection and subsequently proceed to a pathology examination for the gross necropsy. Tissues and organs including the liver, heart, kidneys, spleen, lungs, brain, cerebellum, thymus, adrenals, testicle, epididymis, ovaries, and womb were collected and weighed promptly at necropsy according to the guidelines OECD 407 (2008). The relative organ weights were calculated later. The collected tissues were fixed in neutral buffered formalin solution and tissues from the control and high-dose group (1000 mg/kg) were then embedded in paraffin, sectioned, and stained in hematoxylin and eosin for histopathological examination.

### 2.8. Hematotoxicity Examination In Vitro

Primary mouse peripheral blood mononuclear cells (PBMCs) were isolated from mouse blood through density gradient centrifugation according to the manufacturer’s instructions (Solarbio, Beijing, China) and cultured in RPMI-1640 medium supplemented with 10% fetal bovine serum. PBMCs were treated with curcumol (0, 25, 50, 100 μM) for 36 h, and single-cell suspensions were incubated and stained with PE-anti-CD4 (Biolegend, CA, USA), FITC-anti-CD8a (BD Biosciences, NJ, USA) and PerCP/Cy5.5-anti-CD19 (BD Biosciences, NJ, USA) antibodies for 30 min at room temperature. Then cells were washed and the percentages of different immune cells in PBMCs by flow cytometry were analyzed. 

### 2.9. Statistical Analysis 

Statistical analysis on organ weight, relative organ weight, hematology, and biochemistry were carried out by sex and dosage. Data were present as group mean ± standard deviation for the quantitative results. One-way analysis of variance (ANOVA) was carried out to assess differences in these continuous variables, followed by Kruskal–Wallis test if non-normality. If the *p*-value was less than 0.05, the difference was considered statistically significant. All statistical analysis was conducted by SPSS v26.0 (SPSS Inc., Chicago, IL, USA).

## 3. Results

### 3.1. Dose Formulation Analysis and Pharmacokinetic Assay

Due to the poor water solubility, curcumol was hard to dissolve in water formulation and might interfere with its absorption when administrated orally. Thus, we first investigated the dose formulation of curcumol that ensures its stable suspension or dissolution. Four different formulations were prepared: 50% aqueous olive oil, 50% aqueous 1,2-propylene glycol, deionized water with 10% ethanol and 10% Kolliphor HS15, and deionized water with 10% ethanol and 10% 1,2-propylene glycol. Male SD rats were randomly administered with four formulations orally (1000 mg/kg) followed by a pharmacokinetic (PK) assay.

The main pharmacokinetics parameters of the different dose formulations are calculated and summarized in Table 1. The C_max_ of curcumol was highest in 50% aqueous olive oil, followed by deionized water with 10% ethanol and 10% Kolliphor HS15. Combined with other pharmacokinetics parameter analysis, we found that 50% aqueous olive oil greatly increased the plasma exposure of curcumol as well as extended its clearance time, which dramatically altered the original pharmacokinetic properties of curcumol in vivo. The addition of 10% Kolliphor HS15 in the formulation improved the water solubility of curcumol and remained at a relatively higher plasma level when compared to the others. Hence, we chose deionized water with 10% ethanol and 10% Kolliphor HS15 as our formal dose formulation for repeated toxicity studies.

### 3.2. Clinical Observations

The rats in all the experimental groups survived during the 28 days of the study. There were no abnormalities observed in the rats’ behaviors, skin, fur, and other physical signs from all the groups. The ophthalmologic examination also showed no abnormalities or lesions in all animals on day 29. No other significant clinical changes were observed.

### 3.3. Body Weight and Food Consumption

**Figure 1:**
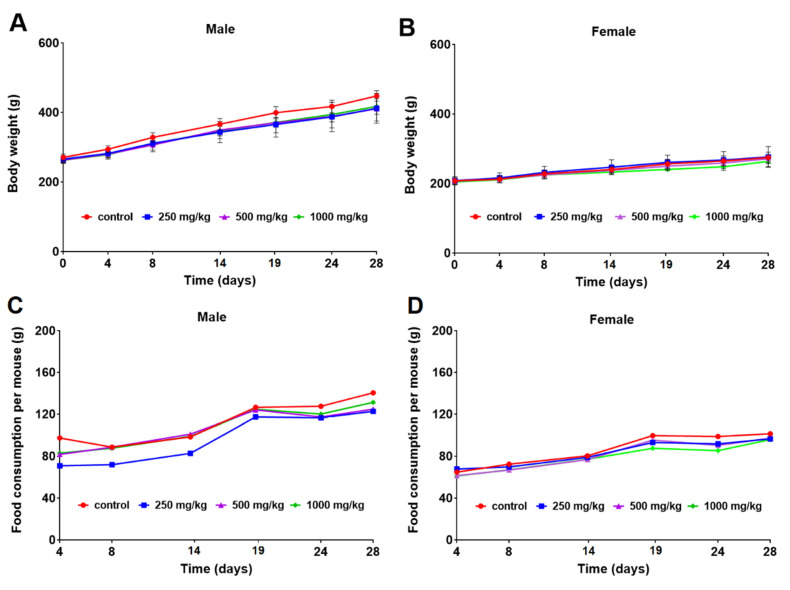
Mean body weights (g) of male (**A**) and female (**B**) rats and food consumption per mouse (g) of male (**C**) and female (**D**) rats orally exposed to 0, 250, 500, and 1000 mg/kg b.w./day curcumol for 28 days.

The mean body weight of the male and female rats is calculated and shown in Figure 1A,B. The body weight increased following a usual pattern of the species during the study period. No statistically significant differences were observed in body weight between the control and curcumol-treated groups (Figure 1A,B). The food consumption of the male and female rats was calculated and shown in Figure 1C,D. The food intake kept generally increasing along with the body weight, without any significant changes in males and females (Figure 1C,D).

### 3.4. Hematology 

The hematology parameters were evaluated after curcumol administration in the rats and provided in Table 2. All variables considered remained unaltered in the female rats after curcumol exposure. However, the WBC count decreased significantly in the male rats of 1000 mg/kg (*p* < 0.05), and the RBC count also decreased remarkably in the male rats exposed to 500 and 1000 mg/kg (*p* < 0.01) in comparison with the control group. In addition, an obvious increase in NEUT (%) was observed in all curcumol-treated groups (*p* < 0.05, *p* < 0.01 and *p* < 0.01, respectively), while the trend of LYMPH (%) was significantly declined in all experimental groups (*p* < 0.05, *p* < 0.01 and *p* < 0.05, respectively). Additionally, the change of HGB, HCT, MCHC, and PLT levels was sporadic, not related to curcumol concentration, and within the normal range for the strain, which was not considered to be of biological relevance [17]. Overall, these data indicated curcumol may have potential hematotoxicity and gender differences. 

### 3.5. Blood Chemistry

Clinical blood biochemistry was measured after curcumol exposure, and the data were summarized in Table 3. Most of these parameters did not alter after repeated exposure to curcumol. The BUN level was significantly decreased in all the female rats exposed to curcumol (*p* < 0.05, *p* < 0.01, and *p* < 0.01, respectively), and the GLU level showed a significant decrease in all the male rats exposed to curcumol in comparison with the control group (*p* < 0.01). Moreover, a significant increase in TB, Na^+^ ions, Cl^−^ ions levels occurred in all the male rats receiving curcumol administration (*p* < 0.01, *p* < 0.01, and *p* < 0.05, respectively). Similarly, an obvious increase in TB, Na^+^ ions levels was observed in the female rats receiving 1000 mg/kg dose (*p* < 0.05), in comparison to the control group. 

### 3.6. Necropsy, Organ Weights and Histopathology

There was no visible pathology observed at the necropsy in the control and curcumol-treated rats for 28 days. Data of absolute organ weights are presented in Table 4. The liver weight decreased significantly in the male rats at low and medium doses (*p* < 0.05), while the spleen weight increased in the female rats at medium and high doses (*p* < 0.01, *p* < 0.05). A significant increase in ovaries was observed in the high-dose group (*p* < 0.05). 

Relative organ weight/brain weight and relative organ weight/b.w. of rats given with curcumol are summarized in Table 5 and Table 6, respectively. In the male rats, the relative liver weight/brain weight ratio decreased (*p* < 0.05) at medium dose, while the relative liver weight/b.w. ratio increased (*p* < 0.05) at the medium dose of the female rats in comparison to the control. In the female rats, the relative spleen weight/brain weight ratio increased significantly at medium and high doses (*p* < 0.01, *p* < 0.05), and the relative spleen weight/b.w. ratio was also increased significantly in the male rats at high doses (*p* < 0.05) and in the female rats at medium and high doses (*p* < 0.01) when compared to the control group. Additionally, the relative organ weight/b.w. ratio for the testicles, epididymis, and ovaries was found to be increased with the statistical differences at medium or high doses, and the change had no apparent relation to the curcumol doses, indicating the change was not considered toxicologically relevant.

**Figure 2:**
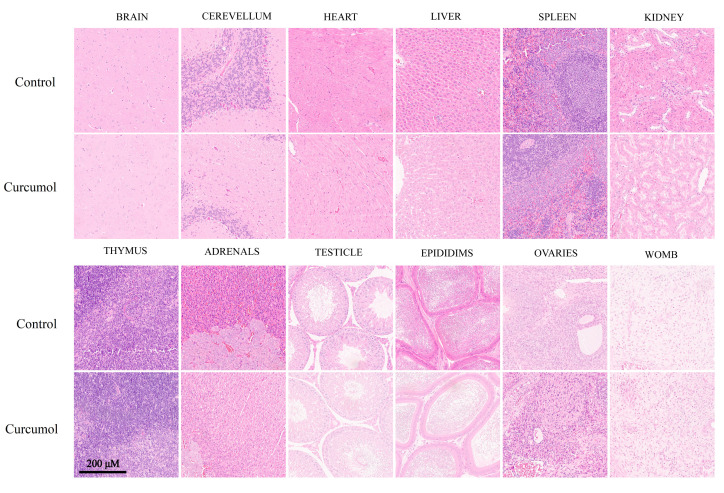
H&E stained sections of brain, cerebellum, heart, liver, spleen, kidney, thymus, adrenals, testicle, epididims, ovaries, and womb from SD rats treated with control and curcumol (1000 mg/kg/day) for 28 days (scale bar: 200 μm).

According to the guideline, tissues from the male and female rats exposed to the 1000 mg/kg dose were submitted for the histopathological examination, and no abnormality or unusual damage was observed in these organs when compared with the control (Figure 2).

### 3.7. Hematotoxicity Study In Vitro

**Figure 3:**
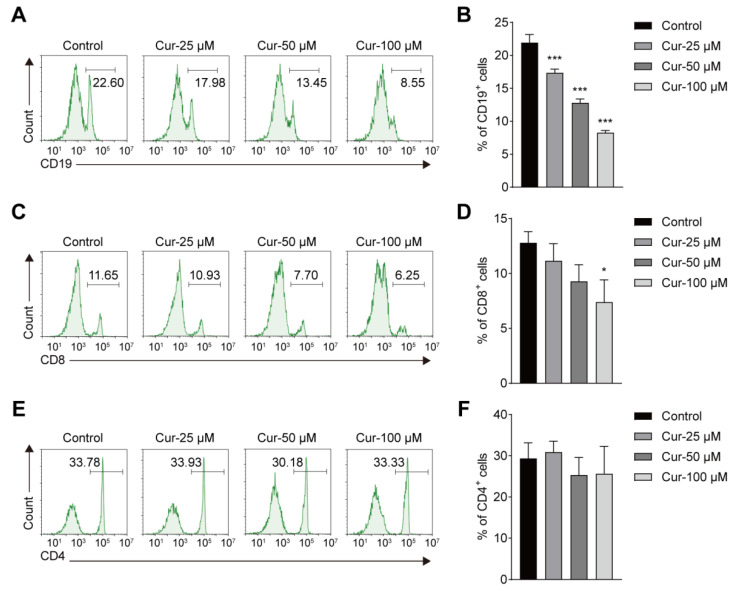
Effects of curcumol on CD19+, CD8+ and CD4+ immune cells in vitro. (**A,C,E**) Flow cytometry analysis of CD19^+^, CD8^+^ and CD4^+^ cells at different dose of curcumol concentrations. (**B,D,F**) Percentages of CD19^+^, CD8^+^ and CD4^+^ cells in peripheral blood mononuclear cells (PBMCs). The data represent the mean ± SD. * *p* < 0.05; *** *p* < 0.001.

Given the potential hematotoxicity of curcumol concluded by the repeated 28-day toxicity study, we further carried out in vitro studies to fully understand the toxicity of curcumol to the hematology. PBMCs were treated with different concentrations of curcumol (0, 25, 50, 100 μM) for 36 h and then detected by flow cytometry. The results showed that the proportion of CD19^+^ B cells and CD8^+^ T cells decreased significantly after curcumol treatment in a dose-dependent manner when compared with the control (Figure 3A–D), while the percentage of CD4^+^ T cells remained unchanged (Figure 3E,F). Consistent with the hematology examination results in the in vivo toxicity study, these data indicated the certain lymphocyte toxicity of curcumol, and further studies are needed to explore the molecular events of curcumol on these lymphocytes.

## 4. Discussion

In the current study, we first investigated and selected deionized water with 10% ethanol and 10% Kolliphor HS15 as the dose formulation of curcumol, which had good solubilization and high absorption to curcumol, and it was then applied to the 28-day repeated toxicity study. Generally, no differences were found in body weight and food consumption in both sexes after oral administration of curcumol to rats for 28 days. However, several hematological parameter (WBC, RBC, NEUT, LYMPH) changes revealed the potential toxic effects in the blood system. In vitro results further confirmed the toxic effect of curcumol on lymphocytes. Some biochemical parameters and organ weight were statistically different in comparison to the control group, but no histopathological changes were found in the related organs studied, indicating these changes were not toxicologically relevant.

With the increasing use of the natural products in the food and pharmaceutical fields, researchers are paying more and more attention to the safety of these active ingredients [18]. Curcumol, a natural product isolated from the traditional Chinese medicine *Rhizoma curcumae*, possesses various therapeutic values for many diseases [19]. However, the low aqueous solubility of curcumol may affect drug absorption in the gastrointestinal tract of animals and further limit the translation of this drug into clinical practice [1,20]. In this case, we explored the dose formulation of curcumol and set up four different formulas for oral administration in rats and followed the pharmacokinetic experiments. The value of AUCs indicated that the addition of olive oil could significantly increase the exposure of curcumol, while its clearance rate decreased. A higher percentage of propylene glycol in the formulation failed to improve the solubility of curcumol and increase the exposure. In addition, it is considered that long-term administration of plant oil may interfere with its judgment of the toxicity of curcumol and its effect on the health of rats [14]. Kolliphor HS15, with good physiological tolerance, has been used as a solubilizer for intravenous and oral drugs [15,21]. In this experiment, the solubilization effect of Kolliphor HS15 on curcumol was significant and increased the exposure of curcumol in vivo compared with that of propylene glycol. Hence, we finally chose deionized water with 10% ethanol and 10% Kolliphor HS15 as the dose formulation in this study.

Due to the wide pharmacological activities of curcumol, the potential clinical indication for the declaration of curcumol has not been determined yet. According to the OECD 407 guidelines, a 28-day study provides information on the effects of repeated oral exposure and can indicate the need for further longer-term studies. The duration of repeated dose toxicity studies depends on the duration of the proposed therapeutic use in humans, and the duration of 28-day toxicity can generally support a clinical development trial up to a 28-day duration and provide rich toxicity data for drug candidates according to the ICH guideline M3(R2). Thus, a 28-day repeated toxicity study of curcumol was performed as preliminary research, providing a basis for subsequent drug discovery of curcumol. For an IND application, it is mandatory to use at least two routes of exposure and two animal species to further elucidate the safety profile of curcumol, and more safety evaluations of curcumol will be carried out in our subsequent work.

Previous studies have shown that curcumol acts on different cells in regulating the signaling pathways. In the liver, curcumol-inhibited fibroblasts proliferate to alleviate liver fibrosis through the protein kinase signaling pathway [22]. Another study showed that curcumol arrested the cell cycle in NHEK cells by downregulating the STAT3 pathway and reducing inflammatory gene expression [23]. Recent network pharmacology prediction suggests that curcumol regulates the cell cycle and immune responses to treat disease [24]. These data indicate that curcumol regulates the cell cycle and participates in the evolution of different cells. However, whether curcumol has a similar effect on immune cells remains unknown. In the current study, some effects were found in our hematology study. At high-dose administration of curcumol (1000 mg/kg), RBC, WBC, and LYMPH levels decreased significantly in the male rats when compared with the control group. Meanwhile, NEUT (%) increased in all treated groups. The changes in the ratio of lymphocytes and neutrophils suggest that the immune and inflammatory levels of the body have changed to some extent [25]. It continues to be meaningful to evaluate the trends of increasing doses. These data suggest that curcumol may have toxic effects on the blood system at concentrations ranging from 250 to 1000 mg/kg. Similar changes were confirmed by PBMCs isolated from mouse blood in vitro (Figure 3). The percentages of CD19^+^ B cells and CD8^+^ T cells decreased markedly when the doses increased. The cultured cells will be included to explore the deep molecular mechanism once we find the target immune cell of curcumol toxicity in the future.

Moreover, some significant changes were found in the absolute organ weights and relative organ weight, including liver, spleen, ovaries, testicle, and epididymis. However, most of them were not dose-dependent and within the normal ranges for this strain, such as the spleen, ovaries, testicles, and epididymis. In the liver, the relative organ weight/body weight ratio was significantly different as well as the liver enzyme levels, ALT, AST, and ALP. Curcumol has been reported to play an important therapeutic role in various liver diseases. Studies have shown that curcumol inhibits the growth and proliferation of hepatocellular carcinoma cells [26]. In addition, curcumol alleviates liver fibrosis by affecting endothelial cell permeability and angiogenesis [7,27]. Studies have shown that the liver is one of the important target organs of curcumol. The results showed that curcumol induced significant changes in ALT, AST, and ALP levels at a high dose of 1000 mg/kg. However, no related histopathologic change was observed in the liver at all dosage groups. Hence, it concluded that curcumol had no apparent hepatic toxicity. 

The levels of the other biochemistry parameters such as TB, Na+, and Cl- were increased significantly after curcumol administration in both sexes, particularly in the high-dose groups. These changes may suggest that the serum bilirubin and ion balance were affected to some extent, but no related histopathological signs were observed. Other parameters, including GLU and BUN, showed no dose response within the selected dosages. No other obvious toxicity was observed till the end of this study.

## 5. Conclusions

In conclusion, the present study conducted a repeated toxicity study of curcumol in male and female rats exposed to different doses of curcumol for 28 days through oral administration for the first time. The rats were well-tolerated to curcumol at the dose of 1000 mg/kg b.w. for 28 days, and no clinical signs of toxicity or change of individual body weight and food consumption were observed in both sexes. Notably, our study revealed potential hematotoxicity of curcumol both in vivo and in vitro. However, the underlying mechanism of the toxicity still deserved further study. In addition, several hematology and biochemistry parameters as well as the weight of organs changed significantly, while no related histopathological signs were observed, suggesting that these changes were considered as not toxicologically relevant.

## Figures and Tables

**Table 1:** Main pharmacokinetic parameters of curcumol with different solvents in rats by oral administration.

Formulation	50% Aqueous Olive Oil	50% Aqueous 1,2-Propylene Glycol	Deionized Water with 10% Ethanol and 10% Kolliphor HS15	Deionized Water with 10% Ethanol and 10% 1,2-Propylene Glycol
No. of rats	2	2	2	2
Dose (mg/kg)	1000	1000	1000	1000
T_max_ (h)	7	1.3	2.1	0.625
C_max_ (ng/mL)	2715	652.5	1915	1025.5
AUC_0-t_ (ug/L·h)	18,637.1	3616.8	10,321.8	4967.1
AUC_0-∞_ (ug/L·h)	18,699.7	3616.8	10,321.8	4977.5
t_1/2_ (h)	2	1	0.8	1.7
CL (L/h/kg)	58.1	322.5	101	206.5
V_Z_ (L/kg)	198.4	451.1	121.7	478.1

**Table 2:** Differential blood cells count data of male and female rats fed with 0, 250, 500, and 1000 mg/kg b.w./day curcumol for 28 days. Values are mean ± SD for five rats/sex/ group.

Male					Female			
No. of Rats	5	5	5	5	5	5	5	5
Dose (mg/kg)	0	250	500	1000	0	250	500	1000
WBC (10^9^/L)	7.07 ± 0.38	6.9 ± 0.51	6.34 ± 1.87	5.65 ± 0.92 *	3.03 ± 0.68	5.42 ± 2.4	3.67 ± 0.62	2.85 ± 1.23
NEUT (%)	11.16 ± 2.14	16.74 ± 3.08 *	20.32 ± 3.33 **	19.9 ± 2.18 **	11.48 ± 3.65	12.4 ± 3.69	15.6 ± 4.2	14.58 ± 2.87
LYMPH (%)	85.68 ± 2.49	80.08 ± 3.33 *	76.58 ± 3.96 **	77.94 ± 2.17 *	86.38 ± 4.02	84.04 ± 4.87	80.88 ± 4.76	82.1 ± 4.46
MONO (%)	2.32 ± 0.19	2.26 ± 0.89	2.18 ± 0.97	1.58 ± 0.64	1.42 ± 0.61	2.46 ± 1.95	2.22 ± 1.4	1.48 ± 0.41
EO (%)	0.84 ± 0.46	0.92 ± 0.27	0.92 ± 0.2	0.58 ± 0.26	0.72 ± 0.24	1.1 ± 0.39	1.3 ± 0.63	1.84 ± 1.33
RBC (10^12^/L)	7.39 ± 0.18	7 ± 0.4	6.83 ± 0.18 **	6.83 ± 0.28 **	6.96 ± 0.35	6.89 ± 0.31	6.85 ± 0.34	6.89 ± 0.36
HGB (g/dL)	14.6 ± 0.56	13.92 ± 1.16	13.34 ± 0.52 **	13.42 ± 0.54 *	13.7 ± 0.54	13.5 ± 0.41	13.18 ± 0.47	13.12 ± 0.44
HCT (%)	40.62 ± 1.73	38.78 ± 2.57	37.44 ± 1.02	38.1 ± 1 *	37.74 ± 1.35	37.8 ± 1.12	36.86 ± 1.17	37.12 ± 1.57
MCV (fL)	54.96 ± 1.68	55.42 ± 2.18	54.88 ± 0.68	55.8 ± 1.8	54.26 ± 2.22	54.88 ± 1.86	53.9 ± 2.11	53.9 ± 1.85
MCH (pg)	19.74 ± 0.67	19.88 ± 0.89	19.54 ± 0.3	19.64 ± 0.51	19.68 ± 0.76	19.6 ± 0.45	19.28 ± 0.63	19.04 ± 0.48
MCHC (g/dL)	35.94 ± 0.51	35.88 ± 0.73	35.62 ± 0.5	35.22 ± 0.52	36.3 ± 0.21	35.7 ± 0.44 *	35.74 ± 0.3 *	35.36 ± 0.78 *
PLT (10^9^/L)	1004.8 ± 91.75	963.4 ± 117.44	955.6 ± 95.54	906.6 ± 20.82 *	1103 ± 69.02	1042.2 ± 135.06	974.6 ± 102.66 *	1052.8 ± 132.87

The significance levels observed are * *p* < 0.05 and ** *p* < 0.01 in comparison to control group values.

**Table 3:** Clinical biochemistry of male and female rats fed with 0, 250, 500, and 1000 mg/kg b.w./day curcumol for 28 days. Values are mean ± SD for five rats/sex/group.

Male					Female			
No. of Rats	5	5	5	5	5	5	5	5
Dose (mg/kg)	0	250	500	1000	0	250	500	1000
TP (g/L)	56.06 ± 2.48	55.18 ± 2.89	53.86 ± 2.08	55.9 ± 2.51	61.7 ± 3.16	59.6 ± 2.78	62.46 ± 6.19	64.98 ± 5.12
ALB (g/L)	39.58 ± 1.77	41.24 ± 2.54	39.68 ± 2.14	41 ± 2.12	47.46 ± 2.17	44.74 ± 2.51	47.84 ± 5.31	50.14 ± 4
ALT (U/L)	38.88 ± 3.77	42.16 ± 6.75	39.68 ± 4.39	44.76 ± 2.09 *	34.7 ± 6.01	35.22 ± 6.92	34.28 ± 3.7	30.98 ± 6.28
AST (U/L)	121.12 ± 14.54	159.48 ± 21.82 *	140.58 ± 11.93 *	125 ± 18.83	114.66 ± 13.75	130.1 ± 15.97	122.1 ± 31.88	98.8 ± 6.81
TB (μmol/L)	1.24 ± 0.27	2.3 ± 0.62 **	3.06 ± 0.69 **	2.58 ± 0.78 **	1.24 ± 0.27	1.56 ± 0.46	1.66 ± 0.34	1.72 ± 0.28 *
ALP (U/L)	163.6 ± 20.48	160.2 ± 43.49	133.4 ± 19.54 *	159 ± 34.9	91.6 ± 11.74	75.4 ± 8.53 *	74.4 ± 22.6	64.4 ± 10.5 **
BUN (mmol/L)	5.38 ± 0.62	5.14 ± 0.19	4.62 ± 0.23 *	5.42 ± 0.61	9.38 ± 1.61	6.84 ± 1.33 *	6.1 ± 0.75 **	6.08 ± 0.7 **
GLU (mmol/L)	8.13 ± 0.8	6.04 ± 0.26 **	6.04 ± 0.36 **	6.24 ± 0.35 **	7.5 ± 0.88	6.48 ± 0.43	7.11 ± 0.28	7.29 ± 0.75
TG (mmol/L)	0.58 ± 0.1	0.66 ± 0.34	0.45 ± 0.2	0.42 ± 0.13	0.4 ± 0.07	0.38 ± 0.09	0.79 ± 0.43	0.3 ± 0.08
TC (mmol/L)	1.44 ± 0.11	1.23 ± 0.22	1.05 ± 0.29 *	1.27 ± 0.28	1.68 ± 0.17	1.57 ± 0.21	1.61 ± 0.16	1.44 ± 0.46
CK (U/L)	953.4 ± 240.91	1159 ± 314.82	1019.6 ± 152.04	773.8 ± 312.7	839.6 ± 278.57	993.6 ± 166.68	1021 ± 393.34	606.2 ± 113.79
K (mmol/L)	4.31 ± 0.21	4.22 ± 0.09	4.43 ± 0.21	4.41 ± 0.13	4.21 ± 0.27	4.18 ± 0.21	4.27 ± 0.32	4.36 ± 0.21
Na (mmol/L)	142.4 ± 0.89	144.8 ± 0.45 **	146.2 ± 1.64 **	147 ± 1.87 **	142.4 ± 0.89	143.8 ± 1.48	144.8 ± 1.48	147.6 ± 1.67 **
Cl (mmol/L)	102.66 ± 0.93	103.42 ± 1.06 **	106.48 ± 1.72 **	106.44 ± 1.19 **	104.32 ± 2.19	104.92 ± 1.05	105.38 ± 2.12	107.58 ± 1.39 *

The significance levels observed are * *p* < 0.05 and ** *p* < 0.01 in comparison to control group values.

**Table 4:** Absolute organ weight of male and female rats fed with 0, 250, 500, and 1000 mg/kg b.w./day curcumol for 28 days. Values are mean ± SD for five rats/sex/group.

Male					Female			
No. of Rats	5	5	5	5	5	5	5	5
Dose (mg/kg)	0	250	500	1000	0	250	500	1000
BRAIN (g)	1.99 ± 0.07	1.87 ± 0.11	2.01 ± 0.14	2.02 ± 0.06	1.85 ± 0.09	1.84 ± 0.09	1.85 ± 0.12	1.91 ± 0.17
HEART (g)	1.44 ± 0.17	1.22 ± 0.16	1.31 ± 0.15	1.29 ± 0.09	0.99 ± 0.21	0.88 ± 0.16	0.89 ± 0.07	0.87 ± 0.04
LIVER (g)	12.72 ± 0.77	11.24 ± 0.88 *	11.11 ± 0.90 *	12.05 ± 1.02	7.51 ± 0.65	8.20 ± 0.70	8.26 ± 0.98	7.64 ± 0.51
SPLEEN (g)	0.91 ± 0.08	0.87 ± 0.08	0.88 ± 0.08	1.04 ± 0.16	0.52 ± 0.05	0.61 ± 0.1	0.66 ± 0.07 *	0.70 ± 0.09 **
KIDNEY (g)	3.02 ± 0.28	2.8 ± 0.21	2.96 ± 0.22	2.89 ± 0.19	1.80 ± 0.21	1.92 ± 0.51	1.75 ± 0.09	1.67 ± 0.13
THYMUS (g)	0.56 ± 0.19	0.51 ± 0.11	0.41 ± 0.09	0.48 ± 0.14	0.41 ± 0.08	0.5 ± 0.12	0.41 ± 0.04	0.39 ± 0.10
ADRENALS (g)	0.04 ± 0.01	0.05 ± 0.01	0.04 ± 0.01	0.05 ± 0.01	0.06 ± 0.01	0.06 ± 0.0	0.06 ± 0.01	0.06 ± 0.01
TESTICLE (g)	3.09 ± 0.36	3.11 ± 0.41	3.20 ± 0.37	3.41 ± 0.25	/	/	/	/
EPIDIDIMS (g)	0.95 ± 0.13	0.94 ± 0.06	1.11 ± 0.18	0.96 ± 0.09	/	/	/	/
OVARIES (g)	/	/	/	/	0.13 ± 0.02	0.14 ± 0	0.15 ± 0.04	0.16 ± 0.01 *
WOMB (g)	/	/	/	/	0.49 ± 0.08	0.64 ± 0.23	0.61 ± 0.17	0.65 ± 0.16

The significance levels observed are * *p* < 0.05 and ** *p* < 0.01 in comparison to control group values.

**Table 5:** Relative organ weight/brain weight of male and female rats fed with 0, 250, 500, and 1000 mg/kg b.w./day curcumol for 28 days. Values are mean ± SD for five rats/sex/group.

Male					Female			
No. of Rats	5	5	5	5	5	5	5	5
Dose (mg/kg)	0	250	500	1000	0	250	500	1000
HEART (%)	72.3 ± 8.4	65.5 ± 10.3	65.0 ± 5.6	64.1 ± 3.5	53.8 ± 11.8	47.4 ± 7.0	48.4 ± 5.3	46 ± 5.3
LIVER (%)	638.5 ± 40.2	604.3 ± 72.4	553.9 ± 49.1 *	597.2 ± 38.0	404.7 ± 24.3	444.8 ± 36.2	444.4 ± 30.9	403.8 ± 48.5
SPLEEN (%)	45.6 ± 3.1	46.9 ± 5.6	43.8 ± 4.8	51.3 ± 7.1	28.2 ± 3.2	32.9 ± 4.8	35.6 ± 3.5 **	37.2 ± 7.6 *
KIDNEY (%)	151.6 ± 12.4	150.7 ± 17.5	147.6 ± 8.3	143.6 ± 7.4	97.1 ± 9.3	103.5 ± 22.4	94.6 ± 6	88.1 ± 7.8
THYMUS (%)	27.8 ± 8.9	27.2 ± 5.4	20.3 ± 5.2	23.8 ± 6.7	21.8 ± 3.9	27.1 ± 5.5	22.3 ± 3.3	20.8 ± 6.6
ADRENALS (%)	2.1 ± 0.7	2.5 ± 0.6	2.1 ± 0.3	2.7 ± 0.5	3.4 ± 0.3	3.5 ± 0.2	3.2 ± 0.5	3.3 ± 0.6
TESTICLE (%)	155.4 ± 22.5	166.6 ± 23.2	159.4 ± 18.4	169.4 ± 13.1	/	/	/	/
EPIDIDIMS (%)	47.7 ± 7.4	50.6 ± 4.0	55.1 ± 8.1	47.7 ± 4.9	/	/	/	/
OVARIES (%)	/	/	/	/	7 ± 1.1	7.4 ± 0.4	7.8 ± 1.8	8.3 ± 1.4
WOMB (%)	/	/	/	/	26.4 ± 4.2	34.4 ± 10.6	33 ± 10.1	34.5 ± 11.1

The significance levels observed are * *p* < 0.05 and ** *p* < 0.01 in comparison to control group values.

**Table 6:** Relative organ weight/body weight of male and female rats fed with 0, 250, 500, and 1000 mg/kg b.w./day curcumol for 28 days. Values are mean ± SD for five rats/sex/group.

Male					Female			
No. of Rats	5	5	5	5	5	5	5	5
Dose (mg/kg)	0	250	500	1000	0	250	500	1000
BRAIN (%)	0.44 ± 0.02	0.46 ± 0.08	0.47 ± 0.03	0.48 ± 0.02	0.66 ± 0.01	0.67 ± 0.06	0.69 ± 0.04	0.75 ± 0.06
HEART (%)	0.32 ± 0.03	0.29 ± 0.01	0.31 ± 0.04	0.31 ± 0.02	0.39 ± 0.09	0.32 ± 0.03	0.33 ± 0.02	0.34 ± 0.03
LIVER (%)	2.88 ± 0.15	2.8 ± 0.09	2.72 ± 0.04	2.94 ± 0.04	2.71 ± 0.11	3.1 ± 0.07	3.01 ± 0.23 *	2.96 ± 0.25
SPLEEN (%)	0.20 ± 0.02	0.22 ± 0.03	0.21 ± 0.01	0.26 ± 0.03 *	0.2 ± 0.02	0.2 ± 0.03	0.24 ± 0.03 **	0.27 ± 0.03 **
KIDNEY (%)	0.69 ± 0.06	0.7 ± 0.03	0.71 ± 0.02	0.69 ± 0.05	0.66 ± 0.04	0.65 ± 0.07	0.66 ± 0.03	0.66 ± 0.05
THYMUS (%)	0.14 ± 0.05	0.11 ± 0.02	0.11 ± 0.02	0.12 ± 0.04	0.15 ± 0.02	0.16 ± 0.03 *	0.15 ± 0.0	0.15 ± 0.03
ADRENALS (%)	0.01 ± 0.0	0.01 ± 0.0	0.01 ± 0.0	0.01 ± 0.0	0.02 ± 0.0	0.02 ± 0.0	0.02 ± 0.0	0.03 ± 0.0
TESTICLE (%)	0.72 ± 0.07	0.77 ± 0.02	0.8 ± 0.05 *	0.81 ± 0.04 **	/	/	/	/
EPIDIDIMS (%)	0.23 ± 0.02	0.24 ± 0.02	0.27 ± 0.05 *	0.22 ± 0.02	/	/	/	/
OVARIES (%)	/	/	/	/	0.05 ± 0.01	0.05 ± 0	0.06 ± 0.01	0.06 ± 0.0 *
WOMB (%)	/	/	/	/	0.17 ± 0.04	0.19 ± 0.03	0.23 ± 0.06	0.25 ± 0.03

The significance levels observed are * *p* < 0.05 and ** *p* < 0.01 in comparison to control group values.

## Data Availability

The data that support the findings of this study are available on request from the corresponding authors.
